# Mitogenome characterization and phylogeny of Huzhu white yak (*Bos grunniens*) in China

**DOI:** 10.1080/23802359.2022.2073835

**Published:** 2022-06-07

**Authors:** Jing Luo, Guangzhen Li, Ruizhe Li, Yongqing Yang, Decang He, Wenxian Liu, Mohammed Yosri, Zhijie Ma

**Affiliations:** a Qinghai Academy of Animal Science and Veterinary Medicine, Qinghai University, Xining, China; b Plateau Livestock Genetic Resources Protection and Innovative Utilization Key Laboratory of Qinghai Province, Xining, China; c Huangzhong Station of Animal Science and Veterinary Medicine in Qinghai Province, Xining, China; d Station of Animal Science and Veterinary Medicine of Huzhu County in Qinghai Province, Haidong, China; e Animal Disease Prevention and Control Center of Menyuan County in Qinghai Province, Haibei, China; f The Regional center for Mycology and Biotechnology, Al Azhar University, Cairo, Egypt

**Keywords:** *Bos grunniens*, mitogneome, assembly, annotation, phylogeny

## Abstract

White yak is a unique and precious economic livestock animal in the world. In this study, the mitogenome of Huzhu white yak was firstly sequenced using Illumina high-throughput sequencing technique and then the assembly was annotated. We also explored mitogenome characterization and phylogeny of Huzhu white yak. Our results showed that the mitogenome of Huzhu white yak is a circular molecule with 16,323bp length including a non-coding control region (D-loop), two ribosomal RNA genes (*12S rRNA* and *16S rRNA*), 22 transfer RNA genes and 13 protein-coding genes. The contents of four nucleotides (A, G, C and T) were 33.71%, 13.21%, 25.80%, and 27.28%, respectively, yielding a lower GC content (39.01%) than AT (60.99%). Phylogenetic analysis suggested that Huzhu white yak possessed the closest relationships with Huanhu, Jiulong, Datong, Jinchuan, Sibu, Ashdan and Pali yak breeds, and closer to wild yak and Bazhou breed.

Yaks (*Bos grunniens*) live in the Qinghai-Tibet Plateau (QTP) and adjacent alpine and subalpine areas (Linnaeus 1766). It has strong adaptability to harsh environments such as high altitude, strong ultraviolet and intense cold and provides local herdsmen with daily necessities such as meat, milk and fur (Wiener et al. 2003). White yak, a unique and precious economic livestock animal in the world, mainly lives in Tianzhu County of Gansu Province and Menyuan, Huzhu and Ledu Counties of Qinghai Province in China (Compilation Committee of animal and poultry records and maps of Qinghai Province 1983). Presently, China own 22 domestic yak breeds including 20 indigenous breeds (Qinghai-Plateau, Huanhu, Xueduo, Yushu, Niangya, Sibu, Pali, Leiwuqi, Tibetan High Mountian, Chawula,Tianzhu, Gannan, Bazhou, Pamier, Zhongdian, Jiulong, Maiwa, Changtai, Jinchuan and Muli) and two improved breeds (Datong and Ashdan) (National Committee of animal genetic resources. 2021). However, except that Tianzhu white yak breed is white, other yak breeds are black. Huzhu white yak (*Bos grunniens*) was firstly described by Li et al (Li et al. 2021), which resides in Tu Autonomous County of Huzhu in Qinghai Province, China. In our recent study, significant maternal genetic difference was detected between Huzhu white yak population and Tianzhu white yak breed based on mtDNA D-loop sequence variations. For instance, we identified 43 and 3 specific haplotypes in Tianzhu white yak and Huzhu white yak, respectively, and significant genetic differentiation was also found between them (*F_ST_*=0.2721, *p* < 0.05) (Li et al. 2021). Therefore, in this study, the whole mitochondrial genome (mitogenome) of Huzhu white yak was firstly assembled and annotated. We also explored the phylogenetic relationship between Huzhu white yak and other yak breeds in China. Our current study would be useful to the genetic resource conservation and molecular breeding programmes of white yak in the future.

Here, an ear sample of Huzhu white yak (*Bos grunniens*) was collected from Tu Autonomous County of Huzhu, Qinghai, China (101°58′N, 36°50′E). The voucher specimen (Sample No.: HZ13-20201023; zhijiema@126.com) is stored in the Key Laboratory of Plateau Livestock Genetic Resources Protection and Innovative Utilization of Qinghai Province, Academy of Animal Science and Veterinary Medicine, Qinghai University (Xining, Qinghai, China). The Illumina NovaSeq 6000 platform was used to sequence the whole genome of Huzhu yak with a sequencing depth of 22.63×.

The mitogenome sequence of Huzhu white yak was submitted to Genbank (Accession No: OK271108). Our results showed that the length of circular mitogenome of Huzhu white yak was 16,323 bp with nucleotides contents as follows: A 33.71%, G 13.21%, C 25.80% and T 27.28%, which yielded a higher AT content (60.99%) than GC content (39.01%). The gene composition, structure and arrangement of mitogenome for Huzhu white yak are similar to that of most mammals (Clayton 2000; Xu et al. 2015; Hao et al. 2016; Hu and Gao, 2016; Niu et al. 2016; Kamalakkannan et al. 2020; Wang et al. 2021). The mitogenome composed of noncoding control region (D-loop), two rRNA subunit genes (*12S rRNA* and *16S rRNA*), 22 tRNA genes and 13 protein coding genes with lengths of 893 bp, 2527 bp, 1511 bp and 11418 bp, respectively (Figure 1, Table 1). The length of the two rRNA genes are 957 bp (*12S rRNA*) and 1570 bp (*16S rRNA*) respectively, which were separated by *tRNA^Phe^*. 22 tRNA genes ranged from 60 bp (*tRNA^ser^*) to 75 bp (*tRNA^Leu^*) and 13 protein coding genes ranged from 201 bp (*ATP8*) to 1830 bp (*ND5*). Among the 13 protein coding genes, ATA is the starting codon of *ND2*, *ND3* and *ND5*, and ATG is the starting codon of others. Three complete stop codons were annotated, i.e. TAA (*ND1*, *COX1*, *COX2*, A*TP8*, *ATP6*, *ND4L*, *ND5* and *ND6*), TAG (*ND2* and *ND3*), AGA (*Cytb*), and two incomplete stop codons were identified, i.e., TA- (*COX3*) and T– (*ND4*) (Table 1). There are four overlaps in the protein-coding genes, including *ATP6* overlaps with *ATP8* for 40 bp, *COX3* overlaps with *ATP6* for 1 bp, *ND4* overlaps with *ND4L* for 7 bp and *ND6* overlaps with *ND5* for 17 bp. Except for 8 tRNA (*Gln, Ala, Asn, Cys, Tyr, Ser, Glu* and *Pro*) and *ND6* genes in light strand, other mitochondrial genes of Huzhu white yak were encoded in heavy strand (Table 1).

**Figure 1.**
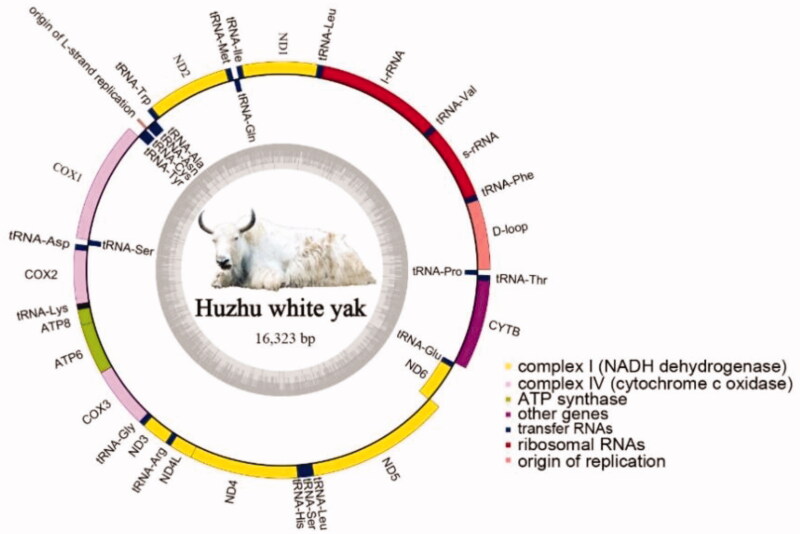
Mitogenome pattern map of Huzhu white yak.

**Table 1.** Mitogenome characterization of Huzhu white yak.

Gene/Region	Position		Size (bp)	Nucleotide composition (%)				Start codon	Stop codon	Strand
	From	To		A (%)	G (%)	C (%)	T (%)			
D-loop	1	893	893	29.35	17.39	23.91	29.35			H
*tRNA^Phe^*	894	960	67	34.33	19.40	22.39	23.88			H
*12S rRNA*	961	1917	957	36.47	18.18	22.57	22.78			H
*tRNA^Val^*	1918	1984	67	38.81	11.94	19.40	29.85			H
*16S rRNA*	1985	3554	1570	38.09	17.01	20.70	24.20			H
*tRNA^Leu^*	3556	3630	75	32.00	17.33	22.67	28.00			H
*ND1*	3633	4589	957	32.39	12.23	29.05	26.33	ATG	TAA	H
*tRNA^Ile^*	4589	4657	69	40.58	15.94	10.14	33.33			H
*tRNA^Gln^*	4655	4726	72	26.39	27.78	9.72	36.11			L
*tRNA^Met^*	4729	4797	69	27.54	18.84	27.54	26.09			H
*ND2*	4798	5841	1044	37.26	8.14	27.20	27.39	ATA	TAG	H
*tRNA^Trp^*	5840	5906	67	37.31	16.42	20.90	25.37			H
*tRNA^Ala^*	5908	5976	69	27.54	23.19	10.14	39.13			L
*tRNA^Asn^*	5978	6051	74	25.68	28.38	14.86	31.08			L
OL	6054	6084	31	38.71	29.03	25.81	6.45			L
*tRNA^Cys^*	6084	6150	67	23.88	26.87	19.40	29.85			L
*tRNA^Tyr^*	6151	6218	68	33.82	20.59	16.18	29.41			L
*COX1*	6220	7764	1545	28.74	16.31	25.44	29.51	ATG	TAA	H
*tRNA^Ser^*	7762	7830	69	24.64	28.99	14.49	31.88			L
*tRNA^Asp^*	7838	7905	68	36.76	17.65	16.18	29.41			H
*COX2*	7907	8590	684	34.36	14.47	22.66	28.51	ATG	TAA	H
*tRNA^Lys^*	8594	8660	67	31.34	20.90	17.91	29.85			H
*ATP8*	8662	8862	201	41.79	5.97	22.89	29.35	ATG	TAA	H
*ATP6*	8823	9503	681	33.33	11.31	26.73	28.63	ATG	TAA	H
*COX3*	9503	10,287	785	26.11	15.16	29.55	29.17	ATG	TA-	H
*tRNA^Gly^*	10,287	10,355	69	31.88	15.94	20.29	31.88			H
*ND3*	10,365	10,712	348	30.17	12.93	28.74	28.16	ATA	TAG	H
*tRNA^Arg^*	10,703	10,771	69	39.13	11.59	10.14	39.13			H
*ND4L*	10,772	11,068	297	31.99	11.78	23.23	33.00	ATG	TAA	H
*ND4*	11,062	12,439	1378	33.38	10.01	27.00	29.61	ATG	T–	H
*tRNA^His^*	12,440	12,509	70	41.43	8.57	15.71	34.29			H
*tRNA^Ser^*	12,510	12,569	60	31.67	16.67	18.33	33.33			H
*tRNA^Leu^*	12,571	12,640	70	37.14	20.00	15.71	27.14			H
*ND5*	12,632	14,461	1830	33.06	10.66	28.85	27.43	ATA	TAA	H
*ND6*	14,445	14,972	528	20.83	29.36	7.58	42.23	ATG	TAA	L
*tRNA^Glu^*	14,973	15,041	69	27.54	21.74	11.59	39.13			L
*Cytb*	15,046	16,185	1140	31.75	13.07	28.86	26.32	ATG	AGA	H
*tRNA^Thr^*	16,189	16,258	70	35.71	15.71	24.29	24.29			H
*tRNA^Pro^*	16,258	16,323	66	24.24	28.79	13.64	33.33			L

Phylogenetic analysis showed that Huzhu white yak possessed the closest relationships with Huanhu, Jiulong, Datong, Jinchuan, Sibu, Ashdan and Pali yak breeds, and was closer to wild yak and Bazhou breed. However, distant genetic relationships were found between Huzhu white yak and the rest of domestic yak breeds (i.e. Maiwa, Xueduo, Zhongdian, Niangya, Qinghai-Plateau, Yushu, Gannan and Tianzhu) (Figure 2). A further extensive survey of yak whole genome in China is warranted to completely clarify the genetic difference and classification between Huzhu white yak and other yak breeds/populations.

**Figure 2.**
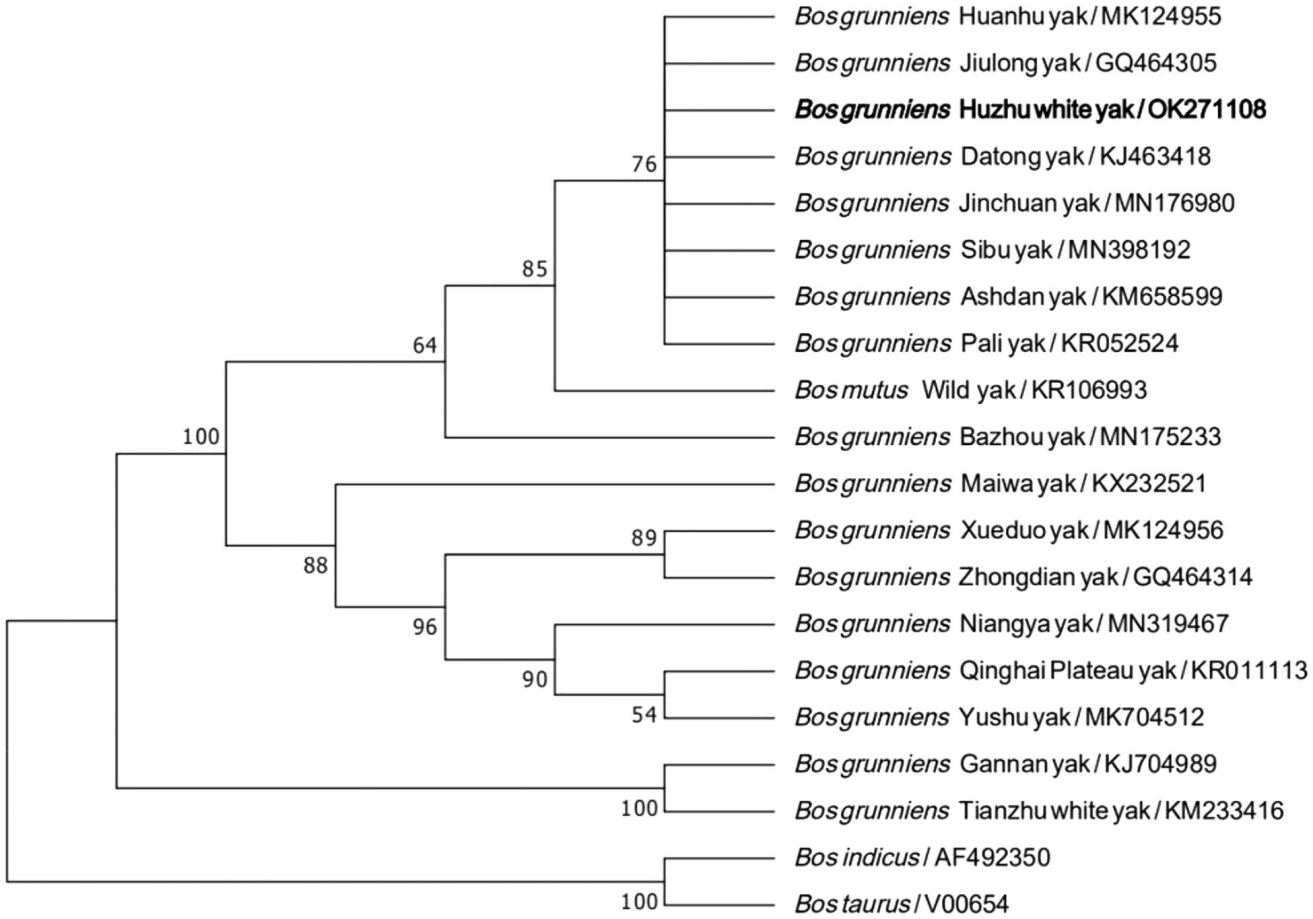
Phylogenetic relationship between Huzhu white yak and 17 yak breeds/populations in China based on mitogenome sequence variations. The support values next to the nodes are based on 1000 bootstrap replicates.

## Ethical approval

This study was conducted with the guidelines of the Council of China and animal welfare requirements. Based on the recommendations of the Regulations for the Administration of Affairs Concerning Experimental Animals of China, the Institutional Animal Care and Use Committee of Qinghai Academy of Animal Science and Veterinary Medicine, Qinghai University approved all animal experiments.

## Author contributions

Zhijie Ma conceived and designed the project. Sample collection personnel include Jing Luo, Guangzhen Li, Ruizhe Li, Zhijie Ma, Decang He, Wenxian Liu and Yongqing Yang. Jing Luo and Zhijie Ma performed the experiment and data analyses. Jing Luo wrote the original manuscript, Zhijie Ma and Mohammed Yosri revised the manuscript. All authors reviewed and approved the final manuscript, submitted the voucher is Jing Luo and Zhijie Ma.

## Data Availability

The genome sequence data that support the findings of this study are openly available in GenBank of NCBI at https://www.ncbi.nlm.nih.gov/ under the accession no. OK271108. The associated BioProject, SRA, and Bio-Sample numbers are PRJNA791839, SRR17319815, and SAMN24365600 respectively.
